# Real-world Effectiveness and Safety of Bictegravir/Emtricitabine/Tenofovir Alafenamide in Comparison With Other Regimens in People With HIV Starting Therapy With AIDS-Defining Conditions: Results From the CoRIS Cohort—The ACTUAS II Study

**DOI:** 10.1093/cid/ciaf162

**Published:** 2025-03-27

**Authors:** Ignacio Pérez-Valero, Diana Corona Mata, Angela Camacho Espejo, Marina Gallo, Alejandro G García-Ruiz de Morales, Chiara Fanciulli, Luz Martín-Carbonero, Sonia Calzado Isbert, Cristina Hernández Gutiérrez, Víctor Asensi, Antonio Rivero Juarez, Antonio Rivero, for the Cohorte de la Red Española de Investigación en SIDA (CoRIS) Cohort, Santiago Moreno, Inma Jarrín, David Dalmau, M Luisa Navarro, M Isabel González, Federico Garcia, Eva Poveda, Jose Antonio Iribarren, Félix Gutiérrez, Rafael Rubio, Francesc Vidal, Juan Berenguer, Juan González, M Ángeles Muñoz-Fernández, Inmaculada Jarrín, Cristina Moreno, Marta Rava, Rebeca Izquierdo, Cristina Marco, Teresa Gómez-García, M Ángeles Muñoz-Fernández, Roxana Juárez, Joaquín Portilla, Irene Portilla, Esperanza Merino, Gema García, Iván Agea, José Sánchez-Payá, Juan Carlos Rodríguez, Livia Giner, Sergio Reus, Vicente Boix, Diego Torrus, Verónica Pérez, Julia Portilla, Héctor Pinargote, María Remedios Alemán, Ana López Lirola, Dácil García, Felicitas Díaz-Flores, M Mar Alonso, Ricardo Pelazas, María Inmaculada Hernández, Lucia Romero, Abraham Bethencourt, Daniel Rodríguez, Víctor Asensi, María Eugenia Rivas-Carmenado, Rebeca Cabo Magadan, Javier Díaz-Arias, Federico Pulido, Rafael Rubio, Otilia Bisbal, M Asunción Hernando, David Rial, María de Lagarde, Adriana Pinto, Laura Bermejo, Mireia Santacreu, Roser Navarro, Juan Martín Torres, José Antonio Iribarren, M José Aramburu, Xabier Camino, Miguel Ángel Goenaga, M Jesús Bustinduy, Harkaitz Azkune, Maialen Ibarguren, Xabier Kortajarena, Ignacio Álvarez-Rodriguez, Leire Gil, Francisco Carmona-Torre, Ana Bayona Carlos, Maialen Lekuona Sanz, Félix Gutiérrez, Catalina Robledano, Mar Masiá, Sergio Padilla, Araceli Adsuar, Rafael Pascual, Marta Fernández, Antonio Galiana, José Alberto García, Xavier Barber, Javier García Abellán, Guillermo Telenti, Lucía Guillén, Ángela Botella, Paula Mascarell, Mar Carvajal, Alba de la Rica, Carolina Ding, Lidia García-Sánchez, Nuria Ena, Leandro López, Jennifer Vallejo, Nieves Gonzalo-Jiménez, Montserrat Ruiz, Christian Ledesma, Santiago López, María Espinosa, Ana Quiles, María Andreo, Roberto Muga, Arantza Sanvisens, Daniel Fuster, Juan Carlos de López Bernaldo de Quirós, Isabel Gutiérrez, Juan Berenguer, Margarita Ramírez, Paloma Gijón, Teresa Aldamiz-Echevarría, Francisco Tejerina, Cristina Diez, Leire Pérez, Chiara Fanciulli, Saray Corral, Joaquín Peraire, Anna Rull, Anna Martí, Consuelo Viladés, Beatriz Villar, Lluïsa Guillem, Montserrat Olona, Graciano García-Pardo, Frederic Gómez-Bertomeu, Verónica Alba, Silvia Chafino, Alba Sánchez, Marta Montero, María Tasias, Eva Calabuig, Miguel Salavert, Juan Fernández, Rosa Blanes, Juan González-García, Ana Delgado-Hierro, José Ramón Arribas, Víctor Arribas, José Ignacio Bernardino, Carmen Busca, Joanna Cano-Smith, Julen Cadiñanos, Juan Miguel Castro, Luis Escosa, Iker Falces, Pedro Herranz, Víctor Hontañón, Alicia González-Baeza, M Luz Martín-Carbonero, Mario Mayoral, Rafael Micán, Rosa de Miguel, Rocío Montejano, Mª Luisa Montes, Luis Ramos-Ruperto, Berta Rodés, Talía Sainz, Elena Sendagorta, Eulalia Valencia, M Del Mar Del Arcos, Alejandro de Gea Grela, Carlos Oñoro López, José Ramón Blanco, Laura Pérez-Martínez, José Antonio Oteo, Valvanera Ibarra, Luis Metola, Mercedes Sanz, Rosa Martínez, Desiré Gil, Álvaro Cecilio, Ruth Caballero, María Aranzazu Caudevilla, David Dalmau, Marina Martinez, Angels Jaén, Mireia Cairó, Javier Martinez-Lacasa, Roser Font, Laura Gisbert, María Rivero, Maider Goikoetxea, María Gracia, Carlos Ibero, Estela Moreno, Jesús Repáraz, Fernando Baigorria, Gemma Navarro, Manel Cervantes Garcia, Sonia Calzado Isbert, Marta Navarro Vilasaro, Ignacio de Los Santos, Alejandro de Los Santos, Lucio García-Fraile, Enrique Martín, Ildefonso Sánchez-Cerrillo, Marta Calvet, Ana Barrios, Azucena Bautista, Carmen Sáez, Marianela Ciudad, Ángela Gutiérrez, María Aguilera García, Santiago Moreno, Santos Del Campo, José Luis Casado, Fernando Dronda, Ana Moreno, M Jesús Pérez, Sergio Serrano-Villar, Mª Jesús Vivancos, Javier Martínez-Sanz, Alejandro Vallejo, Matilde Sánchez-Conde, José Antonio Pérez-Molina, José Manuel Hermida, Erick De La Torre Tarazona, Elena Moreno, Laura Martín Pedraza, Claudio Díaz García, Jorge Díaz, Alejandro García, Raquel Ron, Enrique Bernal, Antonia Alcaraz, Joaquín Bravo, Ángeles Muñoz, Cristina Tomás, Eva Oliver, David Selva, Eva García, Román González, Elena Guijarro, Rodrigo Martínez, María Dolores Hernández, Federico García, Clara Martínez, Leopoldo Muñoz Medina, Marta Álvarez, Natalia Chueca, David Vinuesa, Adolfo de Salazar, Ana Fuentes, Emilio Guirao, Laura Viñuela, Andrés Ruiz-Sancho, Francisco Anguita, Naya Faro, José Peregrina, Lucia Chaves, Marta Illescas, Valme Sánchez, Jorge Del Romero, Montserrat Raposo, Carmen Rodríguez, Teresa Puerta, Juan Carlos Carrió, Mar Vera, Juan Ballesteros, Oskar Ayerdi, Begoña Baza, Eva Orviz, Antonio Antela, Elena Losada, Melchor Riera, María Peñaranda, M Angels Ribas, Antoni A Campins, Mercedes Garcia-Gazalla, Francisco J Fanjul, Javier Murillas, Francisco Homar, Helem H Vilchez, Luisa Martin, Antoni Payeras, Jesús Santos, María López, Cristina Gómez, Isabel Viciana, Rosario Palacios, Luis Fernando López-Cortés, Nuria Espinosa, Cristina Roca, Silvia Llaves, Juan Manuel Tiraboschi, Arkaitz Imaz, María Saumoy, Adrián Curran, Vicenç Falcó, Jordi Navarro, Joaquin Burgos, Paula Suanzes, Jorge García, Vicente Descalzo, Patricia Álvarez, Bibiana Planas, Marta Sanchíz, Lucía Rodríguez, Arnau Monforte, Paola Vidovic, Julián Olalla, Javier Pérez, Alfonso Del Arco, Javier de la Torre, José Luis Prada, Onofre Juan Martínez, Lorena Martinez, Francisco Jesús Vera, Josefina García, Begoña Alcaraz, Antonio Jesús Sánchez Guirao, Álvaro Mena, Berta Pernas, Pilar Vázquez, Soledad López, Brais Castelo, Sofía Ibarra, Guillermo García, Josu Mirena, Oscar Luis Ferrero, Josefina López, Mireia de la Peña, Miriam López, Iñigo López, Itxaso Lombide, Víctor Polo, Joana de Miguel, Beatriz Ruiz Estevez, Maite Ganchegui Aguirre, María Jesús Barberá Gracia, Carlos Galera, Marian Fernández, Helena Albendin, Antonia Castillo, Asunción Iborra, Antonio Moreno, M Angustias Merlos, Inmaculada Chiclano, Concha Amador, Francisco Pasquau, Concepción Gil, José Tomás Algado, Inés Suarez-García, Eduardo Malmierca, Patricia González-Ruano, M Pilar Ruiz, José Francisco Pascual, Luz Balsalobre, Ángela Somodevilla, María de la Villa López, Mohamed Omar, Carmen Herrero, Miguel Alberto de Zárraga, Desirée Pérez, Vicente Estrada, Noemí Cabello, M José Núñez, Iñigo Sagastagoitia, Reynaldo Homen, Ana Muñoz, Inés Armenteros Yeguas, Miguel Górgolas, Alfonso Cabello, Beatriz Álvarez, Laura Prieto, Aws Al-Hayani, Irene Carrillo, José Sanz, Alberto Arranz, Cristina Hernández, María Novella, M José Galindo, Sandra Pérez Gómez, Ana Ferrer, Antonio Rivero Román, Inma Ruíz, Antonio Rivero Juárez, Pedro López, Isabel Machuca, Mario Frias, Ángela Camacho, Ignacio Pérez, Diana Corona, Javier Manuel Caballero, Rafael Rodríguez-Rosado Martinez-Echevarría, Rafael Torres, Juan Macías Sánchez, Pilar Rincón, Luis Miguel Real, Anais Corma, Alejandro González-Serna, Eva Poveda, Alexandre Pérez, Luis Morano, Celia Miralles, Antonio Ocampo, Guillermo Pousada, María Gallego, Jacobo Alonso, Inés Martínez, Carlos Dueñas, Sara Gutiérrez, Marta de la Fuente López, Cristina Novoa, Xjoylin Egües, Pablo Telleria, Carlos Güerri Fernández, Claudia Navarro Valls, Juan Du, Agustin Marcos Blanco, Itziar Arrieta Aldea, Esperanza Cañas Ruan, Cecilia Canepa, Natalia García Giralt

**Affiliations:** Servicio de Enfermedades Infecciosas, Hospital Universitario Reina Sofia, Córdoba, Spain; Grupo de Investigación en Virología Clínica y Zoonosis (GC26), Instituto Maimonides de Investigacion Biomedica de Córdoba, Córdoba, Spain; Departamento de Medicina, Facultad de Medicina y Enfermeria, Universidad de Córdoba, Córdoba, Spain; Centro de Investigación Biomédica en Red de Enfermedades Infecciosas, Instituto de Salud Carlos III, Madrid, Spain; Servicio de Enfermedades Infecciosas, Hospital Universitario Reina Sofia, Córdoba, Spain; Grupo de Investigación en Virología Clínica y Zoonosis (GC26), Instituto Maimonides de Investigacion Biomedica de Córdoba, Córdoba, Spain; Departamento de Medicina, Facultad de Medicina y Enfermeria, Universidad de Córdoba, Córdoba, Spain; Centro de Investigación Biomédica en Red de Enfermedades Infecciosas, Instituto de Salud Carlos III, Madrid, Spain; Servicio de Enfermedades Infecciosas, Hospital Universitario Reina Sofia, Córdoba, Spain; Grupo de Investigación en Virología Clínica y Zoonosis (GC26), Instituto Maimonides de Investigacion Biomedica de Córdoba, Córdoba, Spain; Departamento de Medicina, Facultad de Medicina y Enfermeria, Universidad de Córdoba, Córdoba, Spain; Centro de Investigación Biomédica en Red de Enfermedades Infecciosas, Instituto de Salud Carlos III, Madrid, Spain; Servicio de Enfermedades Infecciosas, Hospital Universitario Reina Sofia, Córdoba, Spain; Grupo de Investigación en Virología Clínica y Zoonosis (GC26), Instituto Maimonides de Investigacion Biomedica de Córdoba, Córdoba, Spain; Departamento de Medicina, Facultad de Medicina y Enfermeria, Universidad de Córdoba, Córdoba, Spain; Centro de Investigación Biomédica en Red de Enfermedades Infecciosas, Instituto de Salud Carlos III, Madrid, Spain; Centro de Investigación Biomédica en Red de Enfermedades Infecciosas, Instituto de Salud Carlos III, Madrid, Spain; Servicio de Enfermedades Infecciosas, Hospital Universitario Ramon y Cajal, Madrid, Spain; Departamento de Medicina, Facultad de Medicina, Universidad de Alcala, Alcala de Henares, Spain; Centro de Investigación Biomédica en Red de Enfermedades Infecciosas, Instituto de Salud Carlos III, Madrid, Spain; Servicio de Microbiología Clínica y Enfermedades Infecciosas, Hospital General Universitario Gregorio Marañón, Madrid, Spain; Grupo de Microbiología Clínica, Enfermedades Infecciosas y SIDA, Instituto de Investigacion Sanitaria Gregorio Marañón, Madrid, Spain; Centro de Investigación Biomédica en Red de Enfermedades Infecciosas, Instituto de Salud Carlos III, Madrid, Spain; Unidad de Enfermedades Infecciosas y VIH, Servicio de Medicina Interna, Hospital Universitario La Paz, Madrid, Spain; Grupo de SIDA y Enfermedades Infecciosas, Instituto de Investigacion Biomedica Hospital La Paz, Madrid, Spain; Servei de Malalties Infeccioses, Parc Taulí Hospital Universitari, Barcelona, Spain; Area de Infecciones e Inmunología, Institut d’Investigacio i Innovacio Parc Taulí, Barcelona, Spain; Departament Medicina, Universitat Autonoma de Barcelona, Sabadell, Spain; Unidad de Enfermedades Infecciosas, Hospital Universitario Príncipe de Asturias, Alcala de Henares, Spain; Servicio de Medicina Interna, Hospital Universitario Central de Asturias, Oviedo, Spain; Servicio de Enfermedades Infecciosas, Hospital Universitario Reina Sofia, Córdoba, Spain; Grupo de Investigación en Virología Clínica y Zoonosis (GC26), Instituto Maimonides de Investigacion Biomedica de Córdoba, Córdoba, Spain; Departamento de Medicina, Facultad de Medicina y Enfermeria, Universidad de Córdoba, Córdoba, Spain; Centro de Investigación Biomédica en Red de Enfermedades Infecciosas, Instituto de Salud Carlos III, Madrid, Spain; Servicio de Enfermedades Infecciosas, Hospital Universitario Reina Sofia, Córdoba, Spain; Grupo de Investigación en Virología Clínica y Zoonosis (GC26), Instituto Maimonides de Investigacion Biomedica de Córdoba, Córdoba, Spain; Departamento de Medicina, Facultad de Medicina y Enfermeria, Universidad de Córdoba, Córdoba, Spain; Centro de Investigación Biomédica en Red de Enfermedades Infecciosas, Instituto de Salud Carlos III, Madrid, Spain

**Keywords:** cohort study, BIC/FTC/TAF, AIDS, naive, effectiveness and safety

## Abstract

**Background:**

The comparative effectiveness and tolerability of bictegravir/emtricitabine/tenofovir alafenamide (BIC/FTC/TAF) has not been sufficiently evaluated in people with AIDS who initiate therapy.

**Methods:**

The aim of the current study was to compare the effectiveness and tolerability of BIC/FTC/TAF with other first-line antiretroviral therapy (ART) regimens in treatment-naive adults from the CoRIS cohort who initiated ART with AIDS. Logistic regression models were used to estimate odds ratios (ORs) of association between initial regimen and achievement of viral suppression (VS), defined as human immunodeficiency virus RNA <50 copies/mL, and immunological recovery (IR), defined as CD4 count >200 cells/μL. Time to VS and the proportion of treatment discontinuations were also evaluated and compared, with all analyses conducted at weeks 24 and 48 after initiation of ART.

**Results:**

We analyzed 90 individuals initiating ART with BIC/FTC/TAF and 94 with other regimens, with similar baseline characteristics. At week 24, BIC/FTC/TAF was associated with a higher proportion of VS (75.6% vs 56.5%; adjusted odds ratio [aOR], 2.78 [95% confidence interval {CI}, 1.28–6.25]) and with a lower proportion of IR (47.7% vs 61.9%; aOR, 0.49 [95% CI, .25–.99]). These differences disappeared by week 48. The proportion of treatment discontinuations was significantly lower with BIC/FTC/TAF than with other regimens (week 24: 4.4% vs 20.2%; week 48: 10% vs 36.2%). At week 48, the main reasons for discontinuations were adverse events (3.3% vs 8.5%), toxicity prevention (1.1% vs 8.5%), ART simplification (0% vs 10.6%), and treatment failure (2.2% vs 4.3%).

**Conclusions:**

In light of our results, BIC/FTC/TAF is an effective and well-tolerated option for starting ART in people with AIDS.

## Teaser

This study compares bictegravir/emtricitabine/tenofovir alafenamide (BIC/FTC/TAF) with other first-line antiretroviral therapies in treatment-naive adults with AIDS-defining conditions. BIC/FTC/TAF showed higher viral suppression rates and lower discontinuation rates at 24 weeks, supporting its efficacy and safety in this population.

## Graphical Abstract

**Graphical Abstract:**
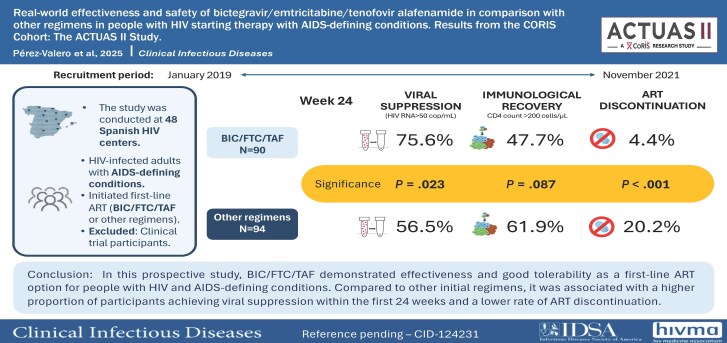

Antiretroviral therapy (ART) has transformed human immunodeficiency virus (HIV) infection into a chronic controllable disease with a life expectancy like that of the general population [1, 2]. However, late diagnosis remains a major problem for many people with HIV (PWH) who are diagnosed at later stages of the disease [3]. A particularly serious situation is the concomitant diagnosis of HIV and AIDS-defining conditions. In this case, it is crucial to choose an ART regimen with a good safety profile that achieves rapid viral suppression (VS) and immunological recovery (IR) [4].

Bictegravir combined with emtricitabine and tenofovir alafenamide (BIC/FTC/TAF) has shown high effectiveness and a good tolerability profile [5–7], comparable to dolutegravir (DTG)–based regimens up to 144 weeks of follow-up [8, 9]. As a result, BIC/FTC/TAF and DTG-based therapies are now preferred first-line therapies for PWH in most therapeutic guidelines [10, 11].

Clinical trials evaluating BIC/FTC/TAF as initial therapy [6, 7], like most studies on other regimens, including those in PWH with advanced disease [12], systematically excluded or did not report data on PWH with AIDS. Moreover, real-world data on this population remain scarce, and no study to date has focused on PWH with AIDS [13–17]. Consequently, it remains unknown whether the high effectiveness and tolerability on BIC/FTC/TAF in the general population extend to PWH diagnosed in the context of an opportunistic disease, especially given that most ART regimens show reduced efficacy/effectiveness in individuals with advanced HIV disease [18–20]. In PWH with AIDS, interactions between ART and treatments for these conditions may lead to either increased or decreased drug exposure, potentially enhancing the development of drug-related toxicities, which can affect tolerability or the therapeutic response to ART [21]. These considerations underscore the importance of evaluating the effectiveness and tolerability of BIC/FTC/TAF compared to other regimens in PWH with AIDS [22].

## METHODS

### Study Design

This study was designed to evaluate the effectiveness and tolerability of BIC/FTC/TAF compared to other first-line ART options in PWH enrolled in the Spanish *Cohorte de la Red de Investigación en SIDA* (CoRIS) cohort, who initiated ART with AIDS between 1 January 2019 and 30 November 2021.

CoRIS is an ongoing, open, multicenter, prospective cohort of adult PWH who were ART naive at study entry, first seen from 1 January 2004, at any of the 48 centers across 14 of Spain's 17 Autonomous Regions. It collects a standardized minimum dataset, including baseline and follow-up sociodemographic, immunological, and clinical data, as well as data on antiretroviral medications. The data undergo periodic quality control procedures to ensure accuracy. Participants are followed up regularly according to routine clinical practice, typically following Grupo de Estudio del SIDA recommendations [11, 23].

### Study Population

We included PWH with AIDS, aged 18 years or older, who initiated first-line ART with BIC/FTC/TAF or other ART regimens between 1 January 2019 and 30 November 2021. For all analyses, we excluded (*i*) individuals who started ART (any regimen) as part of a clinical trial, since treatment selection and patient management in these settings are not fully at the physician's discretion and may not reflect real-life conditions; and (*ii*) those with no follow-up after ART initiation.

### Outcomes

The outcome measures included the proportion of individuals achieving VS, defined as an HIV RNA load <50 copies/mL, at 24 and 48 weeks (±12 weeks) after ART initiation. Other outcomes were (*i*) the proportion of individuals achieving IR, defined as a CD4 count >200 cells/μL, at 24 and 48 weeks (±12 weeks) after ART initiation; (*ii*) time to VS during the first 48 weeks after ART initiation; and (*iii*) the proportion of individuals who discontinued treatment, along with reasons for discontinuation, during the first 24 and 48 weeks of ART. These outcomes were compared between BIC/FTC/TAF and other ART regimens (both as an aggregate group and as individual regimens). Additionally, BIC/FTC/TAF was compared with DTG-based regimens.

Reasons for discontinuation were classified as treatment failure, adverse event (AE), availability of simplified treatment, drug interaction, patient's wish/decision, cost reduction, toxicity prevention, other, and unknown. AEs were categorized as neuropsychiatric, renal, gastrointestinal, skin, liver, and other.

### Statistical Analysis

All participants included in the CoRIS cohort who met the study's selection criteria were analyzed, meaning that outcomes were based on the initial regimen, with any subsequent changes disregarded; thus, once participants started a regimen, they were assumed to have remained on it. Differences in sociodemographic and clinical characteristics based on the initial regimen were assessed using the nonparametric Mann-Whitney test for continuous variables and the χ^2^ test for independence for categorical variables.

Logistic regression models were employed to estimate odds ratios (ORs) for the association between the initial regimen and the achievement of VS and IR at weeks 24 and 48 after ART initiation. For the analysis of time to VS during the first 48 weeks following ART initiation, an individual's follow-up period began at ART initiation and ended at the date of VS, death, last study contact, or after 48 weeks, whichever occurred first. Additionally, for the analyses of VS and IR at 24 and 48 weeks after ART initiation, only cases with available data within the assessment window were included; when multiple measurements were available within that window, the last one recorded was used. We employed the multiple decrement method to calculate the cumulative incidence of VS and used proportional hazards models on the subdistribution hazard to estimate subdistribution hazard ratios (sHRs) for VS, treating deaths before achieving VS as competing events.

Multivariable models were adjusted for the following potential confounders: sex, age at ART initiation, transmission category, educational level, country of origin (Spain, foreign-born), CD4 cell count, and viral load measured within the 6 months prior to ART initiation.

To adjust for the clustering of individuals within centers, robust methods were employed to estimate standard errors, which allowed for the calculation of 95% confidence intervals (CIs) and *P* values. Wald tests were used to derive *P* values.

## RESULTS

Between January 2019 and November 2021, 184 individuals aged 18 years or older began ART with AIDS. Among them, 90 initiated therapy with BIC/FTC/TAF, while 94 started with other regimens (Supplementary Table 1). Baseline characteristics were similar between participants who initiated ART with BIC/FTC/TAF and those who started with other regimens (Table 1). Regarding the distribution of AIDS, it was homogeneous across both groups (Supplementary Table 2), with 1 notable exception: Nearly all individuals with tuberculosis who initiated ART were treated with other regimens due to the contraindication of using BIC/FTC/TAF in patients receiving or about to receive rifampicin.

**Table 1.** Sociodemographic and Clinical Characteristics at Antiretroviral Therapy (ART) Initiation According to Initial ART Regimen, Among Individuals Starting ART With AIDS-Defining Conditions

Characteristic	BIC/FTC/TAF (n = 90)	Other Regimens (n = 94)	*P* Value
Sex			.931
Male	77 (85.6)	80 (85.1)	
Female	13 (14.4)	14 (14.9)	
Age, y			
Median (IQR)	42 (35–54)	43 (35–53)	.633
<30	10 (11.1)	13 (13.8)	.778
30–49	53 (58.9)	51 (54.3)	
≥50	27 (30.0)	30 (31.9)	
Transmission category			.083
Men who have sex with men	53 (58.9)	43 (45.7)	
Heterosexual	30 (33.3)	35 (37.2)	
Intravenous drug use	2 (2.2)	1 (1.1)	
Other/unknown	5 (5.6)	15 (16.0)	
Country of origin			.203
Spain	55 (61.1)	48 (51.1)	
Outside of Spain	34 (37.8)	46 (48.9)	
Unknown	1 (1.1)	0	
CD4 count, cells/μL			
Median (IQR)	58 (26–153)	78 (29–207)	.230
<50	33 (36.7)	28 (29.8)	.553
≥50	41 (45.6)	45 (47.9)	
Unknown	16 (17.8)	21 (22.3)	
Viral load, copies/mL			
Median (IQR)	364 857 (116 000–1 065 259)	347 406 (113 000–775 000)	.572
<100 000	17 (18.9)	17 (18.1)	.785
≥100 000	61 (67.8)	61 (64.9)	
Unknown	12 (13.3)	16 (17.0)	

Data are presented as No. (%) unless otherwise indicated.

Abbreviations: BIC, bictegravir; FTC, emtricitabine; IQR, interquartile range; TAF, tenofovir alafenamide.

### Mortality

During follow-up, a total of 12 deaths were reported with 4 occurring in the BIC/FTC/TAF group and 8 in the other regimens group (*P* = .264). Among the deaths in the BIC/FTC/TAF group, 1 was AIDS related, 1 resulted from a non-AIDS infection, and 2 had an unknown cause. In the other regimens group, 7 deaths were AIDS related, while 1 was due to other causes (Supplementary Table 3).

### Viral Suppression and Immunological Recovery

Viral suppression and immune recovery analyses are presented in Table 2. After 24 weeks from ART initiation, the proportion of individuals who achieved VS was higher with BIC/FTC/TAF compared to other regimens (75.6% vs 56.5%; *P* = .023). However, this difference lost statistical significance at week 48 after ART initiation (87.2% vs 81.6%; *P* = .283). At week 24, the adjusted odds ratio (aOR) for achieving VS with other regimens was 0.36 (95% CI, .16–.78; *P* = .01) times lower than with BIC/FTC/TAF.

**Table 2.** Viral Suppression and Immunological Recovery at 24 and 48 Weeks After Initiation of Antiretroviral Therapy (ART) According to Initial ART Regimen (Intention-to-Treat Analyses)

ART Regimen	24 Weeks			48 Weeks		
	no./No. With Data (%)^a^	Crude OR (95% CI)	Adjusted OR (95% CI)^b^	no./No. With Data (%)^a^	Crude OR (95% CI)	Adjusted OR (95% CI)^b^
Viral suppression						
BIC/FTC/TAF	65/86 (75.6)	1.00	1.00	68/78 (87.2)	1.00	1.00
Other regimens	48/85 (56.5)	0.42 (.20–.89)	0.36 (.16–.78)	62/76 (81.6)	0.65 (.30–1.42)	0.66 (.25–1.74)
*P* value		.023	.010		.283	.402
Immunological recovery						
BIC/FTC/TAF	41/86 (47.7)	1.00	1.00	54/77 (70.1)	1.00	1.00
Other regimens	52/84 (61.9)	1.78 (.92–3.46)	2.03 (1.01–4.05)	62/75 (82.7)	2.03 (.84–4.89)	2.25 (.79–6.42)
*P* value		.087	.046		.114	.130

Abbreviations: ART, antiretroviral therapy; BIC, bictegravir; CI, confidence interval; FTC, emtricitabine; OR, odds ratio; TAF, tenofovir alafenamide.

^a^Number of subjects achieving viral suppression (VS) or immunological recovery (IR), as appropriate/number of subjects with available data on VS or IR, as appropriate (percentage of subjects achieving VS or IR among those with available data).

^b^Adjusted for sex (male, female), age at ART initiation (<30, 30–49, ≥50 years), transmission category (men who have sex with men, heterosexual, other/unknown), educational level (no or compulsory education, secondary or university education, unknown), country of origin (Spain, outside of Spain, unknown), and CD4 count (<50 cells/μL, ≥50 cells/μL, unknown), and viral load (<100 000 copies/mL, ≥100 000 copies/mL, unknown) within the 6 months prior to ART initiation.

In contrast, the proportion of individuals who achieved IR at week 24 after ART initiation was numerically lower with BIC/FTC/TAF than with other regimens (47.7% vs 61.9%; *P* = .087). The aOR of achieving IR with other regimens was 2.03 (95% CI, 1.01–4.05; *P* = .046) times higher than with BIC/FTC/TAF. At week 48 after ART initiation, the proportion of individuals who achieved IR with other regimens was similar than with BIC/FTC/TAF (70.1% vs 82.7%; *P* = .114).

At week 24, the aOR of achieving VS with darunavir/cobicistat/FTC/TAF (DRV/COBI/FTC/TAF) and DTG/lamivudine/abacavir (DTG/3TC/ABC) was 0.16 (95% CI, .04–.63) and 0.29 (95% CI, .10–.83), respectively, both significantly lower (*P* = .008) than with BIC/FTC/TAF (Table 3). Additionally, the aOR for achieving VS with a DTG-based regimen was 0.4 (95% CI, .17–.95; *P* = .037) times lower than with BIC/FTC/TAF. At week 48, the aOR of achieving VS with DTG/3TC/ABC was 0.23 (95% CI, .05–.99; *P* = .049) times lower than with BIC/FTC/TAF. No other significant differences were observed at this timepoint between BIC/FTC/TAF and the other individual regimens, nor between BIC/FTC/TAF and the DTG-based regimens (aOR 0.5 [95% CI, .17–1.47]; *P* = .207). Finally, the risk of achieving IR with the most prevalent ART options among the other regimens was similar to that associated with BIC/FTC/TAF at both week 24 and week 48. The aOR for achieving IR with DTG-based regimens was significantly higher than with BIC/FTC/TAF at week 24 (1.97 [95% CI, 1.03–3.77]; *P* = .042), but this statistical significance was lost at week 48 (1.85 [95% CI, .64–5.34]; *P* = .255).

**Table 3.** Viral Suppression and Immunological Recovery at 24 and 48 Weeks After Initiation of Antiretroviral Therapy (ART) According to Initial ART Regimen (Intention-to-Treat Analyses)

ART Regimen	24 Weeks			48 Weeks		
	no./No. With Data (%)^a^	Crude OR (95% CI)	Adjusted OR (95% CI)^b^	no./No. With Data (%)^a^	Crude OR (95% CI)	Adjusted OR (95% CI)^b^
Viral suppression						
BIC/FTC/TAF	65/86 (75.6)	1.00	1.00	68/78 (87.2)	1.00	1.00
DTG + FTC/TDF	14/26 (53.8)	0.38 (.11–1.34)	0.29 (.08–1.02)	20/24 (83.3)	0.74 (.32–1.68)	0.77 (.29–2.03)
DRV/COBI/FTC/TAF	3/11 (27.3)	0.12 (.04–.41)	0.16 (.04–.63)	8/10 (80.0)	0.59 (.13–2.57)	0.48 (.09–2.51)
DTG/3TC/ABC	9/17 (52.9)	0.36 (.12–1.11)	0.29 (.10–.83)	10/17 (58.8)	0.21 (.07–.65)	0.23 (.05–.99)
Other regimens	22/31 (71.0)	0.79 (.35–1.79)	0.75 (.25–2.24)	24/25 (96.0)	3.53 (.54–22.96)	4.34 (.60–31.26)
*P* value		.004	.008		.011	.049
Immunological recovery						
BIC/FTC/TAF	41/86 (47.7)	1.00	1.00	54/77 (70.1)	1.00	1.00
DTG + FTC/TDF	14/26 (53.8)	1.28 (.50–3.29)	1.40 (.50–3.92)	18/24 (75.0)	1.28 (.45–3.63)	1.24 (.36–4.27)
DRV/COBI/FTC/TAF	7/11 (63.6)	1.92 (.41–8.98)	2.76 (.62–12.30)	8/9 (88.9)	3.41 (.60–19.41)	4.61 (1.30–16.43)
DTG/3TC/ABC	12/17 (70.6)	2.63 (.73–9.56)	3.31 (.89–12.33)	15/17 (88.2)	3.19 (.61–16.68)	3.27 (.50–21.44)
Other regimens	19/30 (63.3)	1.90 (.78–4.62)	1.82 (.56–5.94)	21/25 (84.0)	2.24 (.63–7.88)	2.89 (.61–13.72)
*P* value		.322	.218		.414	.190

Abbreviations: 3TC, lamivudine; ABC, abacavir; ART, antiretroviral therapy; BIC, bictegravir; CI, confidence interval; COBI, cobicistat; DRV, darunavir; DTG, dolutegravir; FTC, emtricitabine; OR, odds ratio; TAF, tenofovir alafenamide; TDF, tenofovir disoproxil fumarate.

^a^Number of subjects achieving viral suppression (VS) or immunological recovery (IR), as appropriate/number of subjects with available data on VS or IR, as appropriate (percentage of subjects achieving VS or IR among those with available data).

^b^Adjusted for sex (male, female), age at ART initiation (<30, 30–49, ≥50 years), transmission category (men who have sex with men, heterosexual, other/unknown), educational level (no or compulsory education, secondary or university education, unknown), country of origin (Spain, outside of Spain, unknown), and CD4 count (<50 cells/μL, ≥50 cells/μL, unknown), and viral load (<100 000 copies/mL, ≥100 000 copies/mL, unknown) within the 6 months prior to ART initiation.

### Time to Viral Suppression

**Figure 1.**
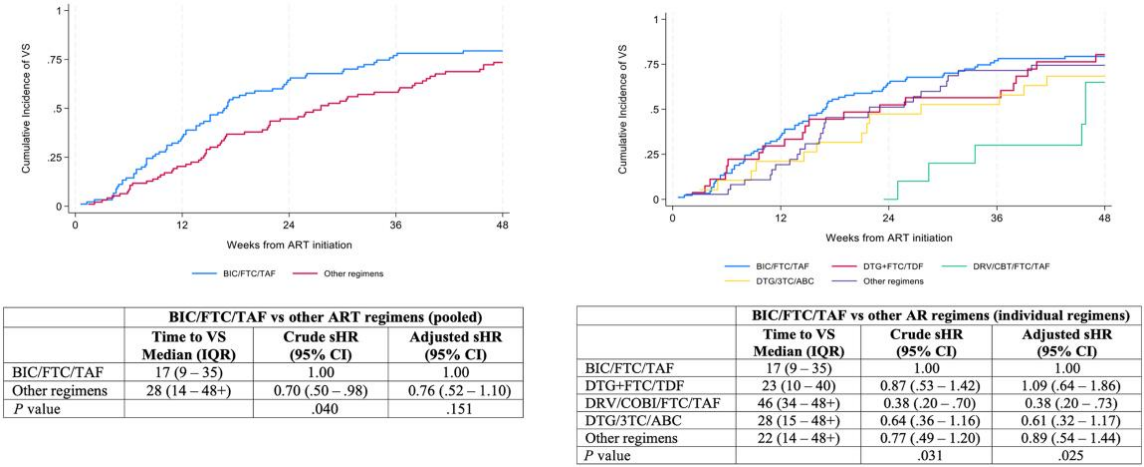
Time to viral suppression during the first 48 weeks after antiretroviral therapy (ART) initiation according to initial ART regimen. Abbreviations: 3TC, lamivudine; ABC, abacavir; ART, antiretroviral therapy; BIC, bictegravir; CI, confidence interval; COBI, cobicistat; DRV, darunavir; DTG, dolutegravir; FTC, emtricitabine; IQR, interquartile range; sHR, subdistribution hazard ratio; TAF, tenofovir alafenamide; TDF, tenofovir disoproxil fumarate; VS, viral suppression.

The median time to VS after ART initiation was 17 weeks (interquartile range [IQR], 9–35 weeks) among individuals who started with BIC/FTC/TAF and 28 weeks (IQR, 14 to >48 weeks) among those who initiated other regimens (Figure 1). Starting ART with other regimens, compared to BIC/FTC/TAF, was associated a lower likelihood of achieving VS (sHR, 0.7 [95% CI, .5–.98]; *P* = .04). However, this difference disappeared after adjusting for potential confounders (adjusted sHR, 0.76 [95% CI, .52–1.1]; *P* = .151).

Time to VS was also compared between BIC/FTC/TAF and the most prevalent ART options among other regimens. Significant differences were found in the time to VS with DRV/COBI/FTC/TAF compared to BIC/FTC/TAF (adjusted sHR, 0.38 [95% CI, .2–.73]; *P* = .025). However, no differences were observed in the time to VS between DTG-based regimens and BIC/FTC/TAF (adjusted sHR, 0.92 [95% CI, .61–1.4]; *P* = .703).

### Treatment Discontinuations

The proportion of treatment discontinuations during the first 24 weeks after ART initiation was significantly lower among individuals starting with BIC/FTC/TAF compared to those initiating other regimens (4.4% vs 20.2%; *P* < .001). This difference is primarily attributed to the lower proportion of discontinuations due to AEs (2.2% vs 8.5%; *P* = .06) and toxicity prevention (main reason: tenofovir disoproxil fumarate withdrawal) (1.1% vs 6.4%; *P* = .06). Discontinuations during the first 48 weeks after ART initiation were similar to those observed in the first 24 weeks (Table 4).

**Table 4.** Treatment Discontinuations During the First 24 and 48 Weeks After Initiation of Antiretroviral Therapy (ART) and Reason for Discontinuation According to Initial ART Regimen

Reason for Discontinuation	BIC/FTC/TAF (n = 90)	No BIC/FTC/TAF					*P* Value
		DTG + FTC/TDF (n = 27)	DRV/COBI/FTC/TAF (n = 11)	DTG/3TC/ABC (n = 19)	Other Regimens^a^ (n = 37)	Total (n = 94)	
During the first 24 weeks							
Treatment changes	4 (4.4)	8 (29.6)	2 (18.2)	1 (5.3)	8 (21.6)	19 (20.2)	.001
Reason for treatment change							
Treatment failure	0	0	0	0	1 (2.7)	1 (1.1)	.318
Adverse event	2 (2.2)	4 (14.8)	2 (18.2)	0	2 (5.4)	8 (8.5)	.059
Simplified treatment available	0	1 (3.7)	0	0	0	1 (1.1)	.318
Drug interaction	1 (1.1)	0	0	1 (5.3)	0	1 (1.1)	1.000
Patient's wish/decision	0	0	0	0	0	0	
Cost reduction	0	0	0	0	0	0	
Toxicity prevention	1 (1.1)	2 (7.4)	0	0	4 (10.8)	6 (6.4)	.060
Other	0	1 (3.7)	0	0	1 (2.7)	2 (2.1)	.167
During the first 48 weeks							
Treatment changes	9 (10.0)	16 (59.3)	2 (18.2)	4 (21.0)	12 (32.4)	34 (36.2)	<.001
Reason for treatment change							
Treatment failure	2 (2.2)	1 (3.7)	0	1 (5.3)	2 (5.4)	4 (4.3)	.423
Adverse event	3 (3.3)	4 (14.8)	2 (18.2)	0	2 (5.4)	8 (8.5)	.136
Simplified treatment available	0	7 (25.9)	0	2 (10.5)	1 (2.7)	10 (10.6)	.001
Drug interaction	2 (2.2)	0	0	1 (5.3)	0	1 (1.1)	.557
Patient's wish/decision	0	0	0	0	0	0	
Cost reduction	0	0	0	0	0	0	
Toxicity prevention^b^	1 (1.1)	3 (11.1)	0	0	5 (13.5)	8 (8.5)	.020
Other	1 (1.1)	1 (3.7)	0	0	2 (5.4)	3 (3.2)	.329

Data are presented as No. (%) unless otherwise indicated.

Abbreviations: 3TC, lamivudine; ABC, abacavir; ART, antiretroviral therapy; BIC, bictegravir; COBI, cobicistat; DRV, darunavir; DTG, dolutegravir; FTC, emtricitabine; TAF, tenofovir alafenamide; TDF, tenofovir disoproxil fumarate.

^a^Other regimens discontinued by week 48: treatment failure (raltegravir [RTG] + FTC/TAF to BIC/FTC/TAF and elvitegravir (EVG)/COBI/FTC/TAF to DRV/COBI + DTG + etravirine), adverse events (efavirenz [EFV]/FTC/TDF to EFV + 3TC/ABC and DTG + FTC/TAF to DTG/3TC), simplification (DTG + FTC/TAF to DTG/3TC) and toxicity prevention, and other (EVG/COBI/FTC/TAF to BIC/FTC/TAF and RTG + FTC/TAF to ART interruption).

^b^Reasons for discontinuation to prevent toxicity: 1 participant on BIC/FTC/TAF interrupted therapy for 1 month to prevent central nervous system toxicity, then resumed treatment. Six participants switched regimens to prevent TDF-related bone and renal toxicity: 2 changed from RTG + FTC/TDF to RTG + FTC/TAF, 1 from DTG + FTC/TDF to DTG/3TC/ABC, 1 from DTG + FTC/TDF to DTG + FTC/TAF, 1 from EFV/FTC/TDF to BIC/FTC/TAF, and 1 interrupted RTG + FTC/TDF before loss to follow-up. One participant simplified therapy from DTG + FTC/TAF to DTG/3TC to avoid potential TAF-related weight gain, while another switched from DTG + FTC/TDF to RTG + FTC/TDF to prevent DTG-associated neurotoxicity.

At week 48, 3 participants discontinued BIC/FTC/TAF due to AEs: 1 experienced impaired renal function and was switched to DTG + rilpivirine (RPV) + 3TC; another developed a moderate headache and switched to doravirine + 3TC/ABC; and the third experienced sensory neuropathy and was switched to DTG/RPV (Table 5).

**Table 5.** Treatment Discontinuations Due to Adverse Events During the First 48 Weeks After Initiation of Antiretroviral Therapy (ART) and Substitution Regimen According to Initial ART Regimen

Adverse Event	BIC/FTC/TAF (n = 90)	No BIC/FTC/TAF					*P* Value
		DTG + FTC/TDF (n = 27)	DRV/COBI/FTC/TAF (n = 11)	DTG/3TC/ABC (n = 19)	Other Regimens (n = 37)	Total (n = 94)	
Any adverse event	3 (3.3)	4 (14.8)	2 (18.2)	0	2 (5.4)	8 (8.5)	.136
Renal	1 (1.1)	1 (3.7)	0	0	2 (5.4)	3 (3.2)	.329
Skin	0	1 (3.7)	2 (18.2)	0	0	3 (3.2)	.087
Gastrointestinal	0	0	0	0	0	0	
Neuropsychiatric	1 (1.1)	1 (3.7)	0	0	0	1 (1.1)	1.000
Liver	0	0	0	0	0	0	
Other	1 (1.1)	1 (3.7)	0	0	0	1 (1.1)	1.000
Substitution regimen	DOR + 3TC/ABC 1 (33.3)	DTG/3TC/ABC 1 (25.0)	DTG/3TC/ABC 1 (50.0)	…	DTG/3TC 1 (50.0)	DTG/3TC/ABC 2 (25.0)	
	DTG/RPV 1 (33.3)	BIC/FTC/TAF 1 (25.0)	RAL + 3TC/ABC 1 (50.0)	…	EFV + 3TC/ABC 1 (50.0)	BIC/FTC/TAF 1 (12.5)	
	DTG + RPV + 3TC 1 (33.3)	DRV/COBI/FTC/TDF 1 (25.0)	…	…	…	DRV/COBI/FTC/TDF 1 (12.5)	
	…	DRV/RTV/FTC/TDF 1 (25.0)	…	…	…	DRV/RTV/FTC/TDF 1 (12.5)	
	…	…	…	…	…	DTG/3TC 1 (12.5)	
	…	…	…	…	…	RAL + 3TC/ABC 1 (12.5)	
	…	…	…	…	…	EFV + 3TC/ABC 1 (12.5)	

Data are presented as No. (%) unless otherwise indicated.

Abbreviations: 3TC, lamivudine; ABC, abacavir; ART, antiretroviral therapy; BIC, bictegravir; COBI, cobicistat; DOR, doravirine; DRV, darunavir; DTG, dolutegravir; EFV, efavirenz; FTC, emtricitabine; RAL, raltegravir; RPV, rilpivirine; RTV, ritonavir; TAF, tenofovir alafenamide; TDF, tenofovir disoproxil fumarate.

### Sensitivity Analyses

Given that nearly all participants with tuberculosis were treated with other regimens, we performed sensitivity analyses excluding these individuals. These analyses showed minimal changes compared to the overall findings: At week 48, the aOR for VS with other regimens lost significance (aOR, 0.68 [95% CI, .23–1.97]; *P* = .475), while the aOR for IR reached significance (aOR, 2.89 [95% CI, 1.09–7.64]; *P* = .032).

## DISCUSSION

In CoRIS, a real-world European cohort, first-line therapy with BIC/FTC/TAF in PWH with AIDS was associated with high proportions of VS and IR at both 24 and 48 weeks, along with low rates of ART discontinuations. Compared to other regimens, BIC/FTC/TAF was associated with higher odds of achieving VS at week 24 (but not at week 48), a shorter time to VS, and a lower rate of ART discontinuations, likely due to a better tolerability profile. Conversely, participants starting ART with BIC/FTC/TAF had lower odds of achieving IR. These results suggest that BIC/FTC/TAF has a favorable tolerability and durability profile in PWH with AIDS, consistent with prior observations in PWH without AIDS.

Since this is the first study to evaluate BIC/FTC/TAF in PWH with AIDS, direct comparisons with previous studies are limited. However, when comparing our results to those of previous studies evaluating BIC/FTC/TAF in individuals with advanced HIV disease, similar rates of VS are observed. Specifically, the rate of VS in participants enrolled in the GS-US-380-1489/1490 clinical trials with advanced HIV disease was 99%, slightly higher than the 87.2% observed in our study [6, 7]. This difference may be due to the exclusion of candidates with AIDS in these 2 trials and the controlled follow-up within a clinical trial setting.

Under real-life conditions, the performance of BIC/FTC/TAF in advanced HIV disease has been evaluated in 3 cohort studies: CoRIS [13], Observational Pharmaco-Epidemiology Research and Analysis (OPERA) [14, 15], and a Taiwanese cohort [16]. All of these studies reported a low rate of ART discontinuations with BIC/FTC/TAF compared to other ART regimens [13–16]. The results in PWH, mostly with advanced disease but without AIDS, were similar to those reported in our study of PWH with AIDS. Our findings support the notion that BIC/FTC/TAF offers greater durability than other regimens, both in individuals with advanced HIV disease, whether or not they have AIDS.

The lower rate of ART discontinuations observed with BIC/FTC/TAF in our study can be attributed to fewer discontinuations due to AEs and to prevent ART-related toxicities. These factors were also associated with a reduced rate of ART discontinuations in the Taiwanese cohort [16], although this association was not observed in the OPERA cohort [14]. This lack of association should be interpreted with caution, as the reasons for discontinuation were unknown for 56% of the participants. Given this, we suggest that the low rate of BIC/FTC/TAF discontinuations due to AEs reported in first-line clinical trials may also apply to PWH with AIDS [6, 7].

In our study, consistent with the OPERA and Taiwanese cohorts [14–16], the likelihood of achieving VS with BIC/FTC/TAF and DTG/3TC/ABC was similar at 48 weeks. Although participants who started with BIC/FTC/TAF achieved VS more quickly than those on DTG/3TC/ABC, the difference was not statistically significant at 48 weeks. This suggests that while BIC/FTC/TAF may provide an advantage in achieving earlier viral suppression, long-term outcomes between these regimens seem comparable. Several factors may explain this, including potential differences in antiviral activity between BIC/FTC/TAF and DTG/3TC/ABC, possibly influenced by the use of TAF or ABC [6]. However, this remains speculative, and adherence differences may also play a role.

Additionally, participants who started therapy with DRV/COBI/FTC/TAF had a lower likelihood of achieving VS at 48 weeks compared to those starting with BIC/FTC/TAF. Further research is needed to confirm whether DRV/COBI/FTC/TAF poses a disadvantage, with results from the Late Presenter Treatment Optimisation Study trial expected to shed light on this [24].

A limitation of BIC/FTC/TAF as first-line therapy in PWH with AIDS is the lower odds of achieving rapid IR compared to other regimens. This finding has not been assessed in previous studies involving advanced HIV disease. In the OPERA cohort, IR rates were reported, but only at 48 weeks after ART initiation. At that timepoint, the rates were comparable between BIC/FTC/TAF (67%) and other regimens (60%–68%) [14]. The slower IR observed in our study up to week 24 may be attributed to selection bias, where BIC/FTC/TAF was chosen for individuals with more advanced disease and poorer immunological status. This hypothesis is supported by the lower CD4 cell counts observed in our study among participants on BIC/FTC/TAF, as well as the fact that the differences in IR were more pronounced when participants with tuberculosis were excluded. This aligns with existing evidence indicating that immune reconstitution in PWH with severe diseases like tuberculosis is generally slower and less effective [25]. Regardless of the validity of this hypothesis, the magnitude of the difference in odds was very small, short-lived, and not associated with worse clinical outcomes (Supplementary Table 3). Therefore, we believe its impact on the selection of BIC/FTC/TAF as a first-line regimen would be minimal.

This study has several strengths. CoRIS is a large prospective cohort that accurately represents the HIV population diagnosed since 2014 in Spain, making the results highly representative of PWH diagnosed with AIDS today. This enables us to obtain an accurate picture of real-world prescription practices and related health outcomes for PWH with opportunistic diseases in our clinical setting.

However, this study also has some limitations. Although the assessment windows for the 24- and 48-week results were broad (±12 weeks), a small percentage of participants lacked data on HIV RNA or CD4 counts during these periods, mainly due to reduced blood draws during the coronavirus disease 2019 pandemic. Another limitation is the relatively narrow eligibility and follow-up period, as BIC/FTC/TAF was approved in Spain in June 2018. Although the number of participants who initiated ART with BIC/FTC/TAF was sufficient to evaluate the study outcomes, future analyses involving a larger participant pool and extended follow-up would provide more robust insights.

Furthermore, we did not analyze immune reconstitution inflammatory syndrome (IRIS) cases, so the impact of IRIS on participants initiating ART with BIC/FTC/TAF or other regimens remains unknown. However, the impact is likely minimal, as no participants discontinued ART due to IRIS.

Finally, as with any observational study, there may be unmeasured biases. Nonetheless, we minimized their impact through rigorous data collection and statistical adjustments.

In conclusion, our study results suggest that BIC/FTC/TAF is a viable first-line therapy for PWH with AIDS. In this population, where rapid viral suppression is crucial, BIC/FTC/TAF demonstrated favorable effectiveness in reducing viral load. While no significant differences in time to VS were observed after adjusting for potential confounders, the regimen showed durability, supported by its favorable safety and tolerability profile.

## Supplementary Material

ciaf162_Supplementary_Data

## Supplementary Data

Supplementary materials are available at *Clinical Infectious Diseases* online. Consisting of data provided by the authors to benefit the reader, the posted materials are not copyedited and are the sole responsibility of the authors, so questions or comments should be addressed to the corresponding author.

## Notes

***Collaborators***

***CoRIS cohort:*** Santiago Moreno, Inma Jarrín, David Dalmau, M Luisa Navarro, M Isabel González, Federico Garcia, Eva Poveda, Jose Antonio Iribarren, Félix Gutiérrez, Rafael Rubio, Francesc Vidal, Juan Berenguer, Juan González, M Ángeles Muñoz-Fernández, Inmaculada Jarrín, Cristina Moreno, Marta Rava, Rebeca Izquierdo, Cristina Marco, Teresa Gómez-García, M Ángeles Muñoz-Fernández, Roxana Juárez, Joaquín Portilla, Irene Portilla, Esperanza Merino, Gema García, Iván Agea, José Sánchez-Payá, Juan Carlos Rodríguez, Livia Giner, Sergio Reus, Vicente Boix, Diego Torrus, Verónica Pérez, Julia Portilla, Héctor Pinargote, María Remedios Alemán, Ana López Lirola, Dácil García, Felicitas Díaz-Flores, M Mar Alonso, Ricardo Pelazas, María Inmaculada Hernández, Lucia Romero, Abraham Bethencourt, Daniel Rodríguez, Víctor Asensi, María Eugenia Rivas-Carmenado, Rebeca Cabo Magadan, Javier Díaz-Arias, Federico Pulido, Rafael Rubio, Otilia Bisbal, M Asunción Hernando, David Rial, María de Lagarde, Adriana Pinto, Laura Bermejo, Mireia Santacreu, Roser Navarro, Juan Martín Torres, José Antonio Iribarren, M José Aramburu, Xabier Camino, Miguel Ángel Goenaga, M Jesús Bustinduy, Harkaitz Azkune, Maialen Ibarguren, Xabier Kortajarena, Ignacio Álvarez-Rodriguez, Leire Gil, Francisco Carmona-Torre, Ana Bayona Carlos, Maialen Lekuona Sanz, Félix Gutiérrez, Catalina Robledano, Mar Masiá, Sergio Padilla, Araceli Adsuar, Rafael Pascual, Marta Fernández, Antonio Galiana, José Alberto García, Xavier Barber, Javier García Abellán, Guillermo Telenti, Lucía Guillén, Ángela Botella, Paula Mascarell, Mar Carvajal, Alba de la Rica, Carolina Ding, Lidia García-Sánchez, Nuria Ena, Leandro López, Jennifer Vallejo, Nieves Gonzalo-Jiménez, Montserrat Ruiz, Christian Ledesma, Santiago López, María Espinosa, Ana Quiles, María Andreo, Roberto Muga, Arantza Sanvisens, Daniel Fuster, Juan Carlos de López Bernaldo de Quirós, Isabel Gutiérrez, Juan Berenguer, Margarita Ramírez, Paloma Gijón, Teresa Aldamiz-Echevarría, Francisco Tejerina, Cristina Diez, Leire Pérez, Chiara Fanciulli, Saray Corral, Joaquín Peraire, Anna Rull, Anna Martí, Consuelo Viladés, Beatriz Villar, Lluïsa Guillem, Montserrat Olona, Graciano García-Pardo, Frederic Gómez-Bertomeu, Verónica Alba, Silvia Chafino, Alba Sánchez, Marta Montero, María Tasias, Eva Calabuig, Miguel Salavert, Juan Fernández, Rosa Blanes, Juan González-García, Ana Delgado-Hierro, José Ramón Arribas, Víctor Arribas, José Ignacio Bernardino, Carmen Busca, Joanna Cano-Smith, Julen Cadiñanos, Juan Miguel Castro, Luis Escosa, Iker Falces, Pedro Herranz, Víctor Hontañón, Alicia González-Baeza, M Luz Martín-Carbonero, Mario Mayoral, Rafael Micán, Rosa de Miguel, Rocío Montejano, Mª Luisa Montes, Luis Ramos-Ruperto, Berta Rodés, Talía Sainz, Elena Sendagorta, Eulalia Valencia, M Del Mar Del Arcos, Alejandro de Gea Grela, Carlos Oñoro López, José Ramón Blanco, Laura Pérez-Martínez, José Antonio Oteo, Valvanera Ibarra, Luis Metola, Mercedes Sanz, Rosa Martínez, Desiré Gil, Álvaro Cecilio, Ruth Caballero, María Aranzazu Caudevilla, David Dalmau, Marina Martinez, Angels Jaén, Mireia Cairó, Javier Martinez-Lacasa, Roser Font, Laura Gisbert, María Rivero, Maider Goikoetxea, María Gracia, Carlos Ibero, Estela Moreno, Jesús Repáraz, Fernando Baigorria, Gemma Navarro, Manel Cervantes Garcia, Sonia Calzado Isbert, Marta Navarro Vilasaro, Ignacio de Los Santos, Alejandro de Los Santos, Lucio García-Fraile, Enrique Martín, Ildefonso Sánchez-Cerrillo, Marta Calvet, Ana Barrios, Azucena Bautista, Carmen Sáez, Marianela Ciudad, Ángela Gutiérrez, María Aguilera García, Santiago Moreno, Santos Del Campo, José Luis Casado, Fernando Dronda, Ana Moreno, M Jesús Pérez, Sergio Serrano-Villar, Mª Jesús Vivancos, Javier Martínez-Sanz, Alejandro Vallejo, Matilde Sánchez-Conde, José Antonio Pérez-Molina, José Manuel Hermida, Erick De La Torre Tarazona, Elena Moreno, Laura Martín Pedraza, Claudio Díaz García, Jorge Díaz, Alejandro García, Raquel Ron, Enrique Bernal, Antonia Alcaraz, Joaquín Bravo, Ángeles Muñoz, Cristina Tomás, Eva Oliver, David Selva, Eva García, Román González, Elena Guijarro, Rodrigo Martínez, María Dolores Hernández, Federico García, Clara Martínez, Leopoldo Muñoz Medina, Marta Álvarez, Natalia Chueca, David Vinuesa, Adolfo de Salazar, Ana Fuentes, Emilio Guirao, Laura Viñuela, Andrés Ruiz-Sancho, Francisco Anguita, Naya Faro, José Peregrina, Lucia Chaves, Marta Illescas, Valme Sánchez, Jorge Del Romero, Montserrat Raposo, Carmen Rodríguez, Teresa Puerta, Juan Carlos Carrió, Mar Vera, Juan Ballesteros, Oskar Ayerdi, Begoña Baza, Eva Orviz, Antonio Antela, Elena Losada, Melchor Riera, María Peñaranda, M Angels Ribas, Antoni A Campins, Mercedes Garcia-Gazalla, Francisco J Fanjul, Javier Murillas, Francisco Homar, Helem H Vilchez, Luisa Martin, Antoni Payeras, Jesús Santos, María López, Cristina Gómez, Isabel Viciana, Rosario Palacios, Luis Fernando López-Cortés, Nuria Espinosa, Cristina Roca, Silvia Llaves, Juan Manuel Tiraboschi, Arkaitz Imaz, María Saumoy, Adrián Curran, Vicenç Falcó, Jordi Navarro, Joaquin Burgos, Paula Suanzes, Jorge García, Vicente Descalzo, Patricia Álvarez, Bibiana Planas, Marta Sanchíz, Lucía Rodríguez, Arnau Monforte, Paola Vidovic, Julián Olalla, Javier Pérez, Alfonso Del Arco, Javier de la Torre, José Luis Prada, Onofre Juan Martínez, Lorena Martinez, Francisco Jesús Vera, Josefina García, Begoña Alcaraz, Antonio Jesús Sánchez Guirao, Álvaro Mena, Berta Pernas, Pilar Vázquez, Soledad López, Brais Castelo, Sofía Ibarra, Guillermo García, Josu Mirena, Oscar Luis Ferrero, Josefina López, Mireia de la Peña, Miriam López, Iñigo López, Itxaso Lombide, Víctor Polo, Joana de Miguel, Beatriz Ruiz Estevez, Maite Ganchegui Aguirre, María Jesús Barberá Gracia, Carlos Galera, Marian Fernández, Helena Albendin, Antonia Castillo, Asunción Iborra, Antonio Moreno, M Angustias Merlos, Inmaculada Chiclano, Concha Amador, Francisco Pasquau, Concepción Gil, José Tomás Algado, Inés Suarez-García, Eduardo Malmierca, Patricia González-Ruano, M Pilar Ruiz, José Francisco Pascual, Luz Balsalobre, Ángela Somodevilla, María de la Villa López, Mohamed Omar, Carmen Herrero, Miguel Alberto de Zárraga, Desirée Pérez, Vicente Estrada, Noemí Cabello, M José Núñez, Iñigo Sagastagoitia, Reynaldo Homen, Ana Muñoz, Inés Armenteros Yeguas, Miguel Górgolas, Alfonso Cabello, Beatriz Álvarez, Laura Prieto, Aws Al-Hayani, Irene Carrillo, José Sanz, Alberto Arranz, Cristina Hernández, María Novella, M José Galindo, Sandra Pérez Gómez, Ana Ferrer, Antonio Rivero Román, Inma Ruíz, Antonio Rivero Juárez, Pedro López, Isabel Machuca, Mario Frias, Ángela Camacho, Ignacio Pérez, Diana Corona, Javier Manuel Caballero, Rafael Rodríguez-Rosado Martinez-Echevarría, Rafael Torres, Juan Macías Sánchez, Pilar Rincón, Luis Miguel Real, Anais Corma, Alejandro González-Serna, Eva Poveda, Alexandre Pérez, Luis Morano, Celia Miralles, Antonio Ocampo, Guillermo Pousada, María Gallego, Jacobo Alonso, Inés Martínez, Carlos Dueñas, Sara Gutiérrez, Marta de la Fuente López, Cristina Novoa, Xjoylin Egües, Pablo Telleria, Carlos Güerri Fernández, Claudia Navarro Valls, Juan Du, Agustin Marcos Blanco, Itziar Arrieta Aldea, Esperanza Cañas Ruan, Cecilia Canepa, Natalia García Giralt.

***Acknowledgment*. CoRIS cohort:**

***Ethics approval and patient consent.*** The CoRIS cohort was approved by the Clinical Research Ethics Committee (CREC) of Gregorio Marañón General University Hospital. All patients provided written informed consent to participate in CoRIS. This study was approved by the CREC of Córdoba on 30 June 2021 (reference number 5096).

***Disclaimer.*** Gilead Sciences had no role in the study design, data collection, analysis, interpretation, or writing of the manuscript.

***Financial support.*** This work was supported by an investigator-sponsored research grant from Gilead Sciences (grant number IN-ES-380-6277 to A. R.). The CoRIS cohort is funded by the Instituto de Salud Carlos III (CB21/13/00091).

***Potential conflicts of interest**.* I. P.-V., L. M. C., and A. R. report personal fees for speakers bureaus and advisory boards from ViiV Healthcare, Janssen-Cilag, Gilead Sciences, and MSD. I. P.-V. and A. R. also report travel grants from Gilead Sciences and Janssen-Cilag, and their institution (Instituto Maimonides de Investigacion Biomedica de Córdoba) received a research grant from Gilead Sciences to conduct this study. D. C. M., A. C. E., and M. G. report personal fees from ViiV Healthcare. A. G. G.-R. d. M. reports personal fees from ViiV Healthcare; nonfinancial support from ViiV Healthcare, Janssen-Cilag, and Gilead Sciences; and research and travel grants from Gilead Sciences, outside the submitted work. All other authors report no potential conflicts.
