# Supplementary material for: Real-world Effectiveness and Safety of Bictegravir/Emtricitabine/Tenofovir Alafenamide in Comparison With Other Regimens in People With HIV Starting Therapy With AIDS-Defining Conditions: Results From the CoRIS Cohort—The ACTUAS II Study
Source: Clin Infect Dis. 2025 Mar 27;81(4):e93–e101. doi: 10.1093/cid/ciaf162 (PMC12596416; doi:10.1093/cid/ciaf162)
Supplement: ciaf162_Supplementary_Data [file ciaf162_supplementary_data.zip › Table S1.docx]

**Table S1: Description of initial ART regimens prescribed* between January 1, 2019 and November 30, 2021**

| **Initial ART regimen**  **N = 184** | **N (%)** |
| --- | --- |
| Bictegravir/emtricitabine/tenofovir alafenamide  Dolutegravir + emtricitabine/tenofovir disoproxil fumarate  Dolutegravir/lamivudine/abacavir  Darunavir/cobicistat/emtricitabine/tenofovir alafenamide  Dolutegravir/lamivudine  Dolutegravir + emtricitabine/tenofovir alafenamide  Efavirenz/emtricitabine/tenofovir disoproxil fumarate  Raltegravir + emtricitabine/tenofovir disoproxil fumarate  Elvitegravir/cobicistat/emtricitabine/tenofovir alafenamide  Raltegravir + emtricitabine/tenofovir alafenamide  Raltegravir + lamivudine/abacavir | 90 (48.9)  27 (14.7)  19 (10.3)  11 (6.0)  9 (4.9)  8 (4.3)  8 (4.3)  6 (3.3)  3 (1.6)  2 (1.1)  1 (0.5) |

* The treatment prescription was decided based on the characteristics of each patient/case by the responsible physician, according to their clinical judgment and following the recommendations established at the time in the GESIDA clinical guidelines for the management of antiretroviral treatment.
