# Supplementary material for: Real-world Effectiveness and Safety of Bictegravir/Emtricitabine/Tenofovir Alafenamide in Comparison With Other Regimens in People With HIV Starting Therapy With AIDS-Defining Conditions: Results From the CoRIS Cohort—The ACTUAS II Study
Source: Clin Infect Dis. 2025 Mar 27;81(4):e93–e101. doi: 10.1093/cid/ciaf162 (PMC12596416; doi:10.1093/cid/ciaf162)
Supplement: ciaf162_Supplementary_Data [file ciaf162_supplementary_data.zip › Table S2.docx]

**Table S2. Description of the AIDS-defining conditions present at ART initiation**

|  | **BIC/FTC/TAF**  **N = 115*** | **Other regimens**  **N = 116*** |
| --- | --- | --- |
| Pneumocystis pneumonia, n (%) | 29 (25.2) | 32 (27.6) |
| Candidiasis (oesophageal or pulmonary), n (%) | 21 (18.3) | 14 (12.1) |
| HIV Wasting Syndrome, n (%) | 19 (16.5) | 10 (8.6) |
| Kaposi Sarcoma, n (%) | 14 (12.2) | 14 (12.1) |
| Mycobacterium tuberculosis (pulmonary and extrapulmonary), n (%) | 1 (0.9) | 18 (15.51) |
| Disseminated CMV infection, n (%) | 7 (6.1) | 7 (6.0) |
| Brain toxoplasmosis, n (%) | 6 (5.2) | 6 (5.2) |
| Non-Hodgkin Lymphoma, n (%) | 2 (1.7) | 5 (4.3) |
| Extrapulmonary histoplasmosis, n (%) | 4 (3.5) | 2 (1.7) |
| Cytomegalovirus chorioretinitis, n (%) | 1 (0.9) | 2 (1.7) |
| HIV encephalopathy, n (%) | 1 (0.9) | 2 (1.7) |
| Progressive multifocal leukoencephalopathy, n (%) | 2 (1.7) | 0 (0) |
| Extrapulmonary cryptococcosis, n (%) | 2 (1.7) | 0 (0) |
| HSV pneumonitis or esophagitis, n (%) | 2 (1.7) | 0 (0) |
| Cryptosporidiosis, n (%) | 1 (0.9) | 1 (0.9) |
| Mycobacterium. avium complex, n (%) | 1 (0.9) | 1 (0.9) |
| Salmonella bacteriemia or multiple recurrent bacterial infections, n (%) | 2 (1.7) | 2 (1.7) |

*** BIC/FTC/TAF: 70 participants initiated therapy with one, 15 with two and 5 with three AIDS-defining conditions. Other regimens: 79 participants initiated therapy with one, 9 with two, 5 with three and 1 with four AIDS-defining conditions.**
