# Supplementary material for: Real-world Effectiveness and Safety of Bictegravir/Emtricitabine/Tenofovir Alafenamide in Comparison With Other Regimens in People With HIV Starting Therapy With AIDS-Defining Conditions: Results From the CoRIS Cohort—The ACTUAS II Study
Source: Clin Infect Dis. 2025 Mar 27;81(4):e93–e101. doi: 10.1093/cid/ciaf162 (PMC12596416; doi:10.1093/cid/ciaf162)
Supplement: ciaf162_Supplementary_Data [file ciaf162_supplementary_data.zip › Table S3.docx]

**Table S3: Selection of the study population for the ITT analyses of VS and IR at 24 and 48 weeks after ART initiation**

|  | **At 24 (±12) weeks** | | | **At 48 (±12) weeks** | | |
| --- | --- | --- | --- | --- | --- | --- |
|  | **Total**  **N = 184** | **BIC/FTC/TAF**  **N = 90** | **Other regimens**  **N = 94** | **Total**  **N = 184** | **BIC/FTC/TAF**  **N = 90** | **Other regimens**  **N = 94** |
| **Overall exclusions**  Death before the assessment window  AIDS-related death  Death to non-AIDS infection  Death due to other causes  Unknown cause of death  Last visit to the centre before the assessment window  No visit in the assessment window  **Exclusions for Viral suppression analyses**  Available visit in the assessment window but missing viral load  **ITT analyses**  **Exclusions for Immunological recovery analyses**  Available visit in the assessment window but missing CD4  **ITT analyses** | 4 (2.2)  3 (1.6)  0  1 (0.5)  0  3 (1.6)  1 (0.5)  5 (2.7)  **171 (92.9)**  6 (3.3)  **170 (92.4)** | 0  0  0  0  0  0  0  4 (4.4)  **86 (95.6)**  4 (4.4)  **86 (95.6)** | 4 (4.3)  3 (3.2)  0  1 (1.1)  0  3 (3.2)  1 (1.1)  1 (1.1)  **85 (90.4)**  2 (2.1)  **84 (89.4)** | 12 (6.5)  8 (4.3)  1 (0.5)  1 (0.5)  2 (1.0)  5 (2.7)  7 (3.8)  6 (3.3)  **154 (83.7)**  8 (4.3)  **152 (82.6)** | 4 (4.4)  1 (1.1)  1 (1.1)  0  2 (2.2)  2 (2.2)  3 (3.3)  3 (3.3)  **78 (86.7)**  4 (4.4)  **77 (85.6)** | 8 (8.5)  7 (7.4)  0  1 (1.1)  0  3 (3.2)  4 (4.3)  3 (3.2)  **76 (80.9)**  4 (4.3)  **75 (79.8)** |
